# A New Tool for Breast Anthropometric Measurements: Presentation and Validation for Women and Men

**DOI:** 10.1007/s00266-019-01467-6

**Published:** 2019-08-01

**Authors:** Maksym Mikołajczyk, Anna Kasielska-Trojan, Bogusław Antoszewski

**Affiliations:** Plastic, Reconstructive and Aesthetic Surgery Clinic, Medical University of Łódź, Kopcinskiego 22, 90-153 Lodz, Poland

**Keywords:** Anthropometry, Breast, Linear measurement, Web application

## Abstract

**Introduction:**

Anthropometric measurements of breasts are crucial for planning surgical procedures; however, there are no practical solutions for their quick, digital performance. The aim of the study was to present and validate a self-designed web application BreastIdea (BI) designed for indirect breast anthropometry.

**Methods:**

Ten male and 10 female volunteers had their chests measured directly according to the routine clinical practice. Then their chests were photographed in non-standardised conditions. Corresponding measurements were performed using BI. Accuracy and both relative and absolute reliability of BI measurements were investigated.

**Results:**

Breast assessments using BI yielded highly accurate results and presented near-perfect precision when compared to direct anthropometric measurements of the breast. Indirect anthropometry eliminates the necessity to trace the body’s curves, which usually introduces a bias to linear measurements.

**Conclusion:**

BI web application is a reliable tool for indirect breast measurements in a clinical setting, providing accurate results regardless of chest pathology and photograph standardisation.

**Level of Evidence IV:**

This journal requires that authors assign a level of evidence to each article. For a full description of these Evidence-Based Medicine ratings, please refer to the Table of Contents or the online Instructions to Authors www.springer.com/00266.

## Introduction

Planning a surgical procedure on the breast, be it reconstructive, reductive, oncological or aesthetic, breast measurements prove indispensable for state-of-the-art surgery [1]. Proportions, asymmetries and levels play a major role in planning and optimising treatment results. Preoperative chest evaluation is also crucial to accurate breast implant choices for aesthetic and reconstructive reasons [2].

Even though the consistency of outcome of manual measurements is highly dependent on the measurer’s skills and experience, as well as available precision of the tools used, those crucial measurements are mostly done by hand (direct anthropometry). The female breast is one of the most difficult organs to assess objectively [2]. Not only size, but also contour, volume and asymmetry need to be considered [3]. Some breast surgeons use 3D scanning, which would yield accurate results. The technology is not yet as accessible in clinical practice as a simple tape measure. What is more, it is time-consuming and requires expensive devices and a trained specialist to acquire and analyse the scan [4]. There have been attempts to perform breast measurements on photographs using graphics software packages [5]. However, the studies were based on photographs captured in standardised conditions, which are not possible to replicate in clinical settings. What is more, the software used was not clinically oriented.

Due to the lack of freely accessible and easy to use tools for indirect breast anthropometry, we decided to design such software. The tool was not created as an alternative to direct measurements, which remain a gold standard in breast surgeons’ clinical practice. Photograph-based breast analysis may, however, be helpful as an additional source of clinical data. It may help to extract metric data from the image, e.g. while consulting the patient with other surgeons and assessing asymmetry. Moreover, it may serve as a valuable tool for scientific research, like metric evaluation of breast asymmetry performed by independent observers without the need for personal contact with the patient.

Our aim is to present the self-designed web application—BreastIdea (BI)—a tool for linear breast measurements, allowing to substitute a physical pen and tape measure with their digital counterparts, thus increasing the objectivity of breast assessment. In the article, we show the process of its validation, as well as some pitfalls to be avoided by potential users.

## Methods

The protocol of the study was approved by the Bioethics Committee of the Medical University (RNN/100/18/KE). Written informed consent was obtained from all subjects. The study consisted of three phases. The first was web application design and development, the second—direct breast measurements and the third—digital measurements using BI (indirect breast measurements). The measurements performed with BI were compared with direct measurements performed by a plastic surgeon.

The study groups consisted of 10 males, aged 18–37 (mean age 23.7 years, SD 5.04 years) and 10 females, aged 18–45 (mean age 29.6 years, SD 9.38). The volunteers were randomly selected from students of the Medical University and from patients at the Plastic, Reconstructive and Aesthetic Surgery Clinic, University Hospital, regardless of age (over 18 years old), breast size, breast ptosis and deformation (e.g. Poland syndrome). In the female group, 6 women (volunteers: students, patients) had natural breasts and 4 were clinic patients after breast surgery [breast reduction and lift (*n* = 2), breast enlargement (*n* = 1), breast symmetrisation due to Poland syndrome (*n* = 1)]. None of the male participants had chest surgery; however, two had mild lipomastia. The exclusion criterion for the female and male groups was obesity—body mass index above 30. The study process was identical for all patients.

### Web Application Design and Development

**Fig. 1:**
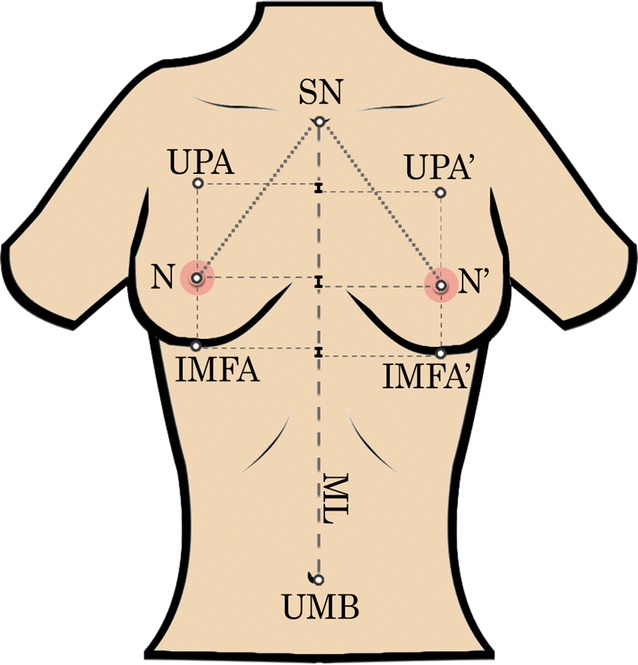
Illustration of all the chosen landmarks and measurements. ’ Left, *SN* sternal notch, *UPA* upper pole apex, *N* nipple, *IMFA* inframammary fold apex, *ML* medial line, *UMB* umbilicus

Our initial assumption was to design an application that was easy to use and access (through a web browser). The user interface was written in Hypertext Markup Language (HTML) with Cascading Style Sheets (CSS), while the logic was supplied by JavaScript (JS)—a standard in web development [6]. Seven plastic surgeons specialising in breast surgery were asked to identify clinically relevant breast measurements. The following linear breast parameters were chosen: the difference in the levels of nipples, medial line–nipple distance (right and left), sternal notch–nipple distance (right and left), the difference in the levels of inframammary folds apices, medial line–inframammary fold apex distance (right and left), inframammary fold apex–nipple distance (right and left), the difference in the levels of both upper pole apices, and upper pole apex–nipple distance (right and left) (Fig. 1). The parameters were not gender specific, applying to both males and females.

For aesthetic assessment, we added proportions of the upper to the lower pole as well as medial to lateral part, derived from the measurements listed above. Following Mallucci et al., for vertical parameters we used the ratio of distance from upper pole apex to nipple and distance from inframammary fold apex to nipple (with the most appealing proportion of 45:55) [7]. For horizontal parameters, we used the ratio of distance from the breast’s lateral border to the nipple and distance from the breast’s medial border to the nipple (with most appealing proportion of 40:60), as suggested by Lewin et al. [8].

**Fig. 2:**
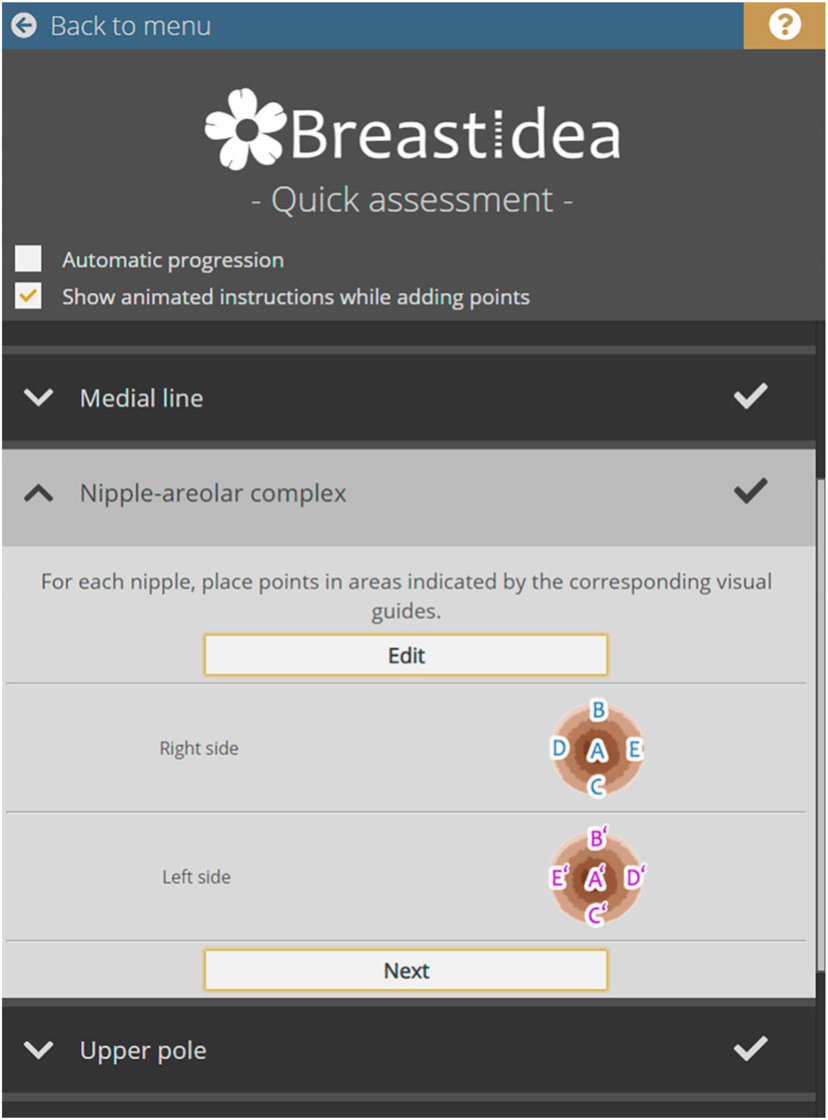
The overview of the user interface. The instructions depend on assessment phase, and they update dynamically

BI guides the user through the process of digital breast measurements. Each measurement consists of placing a number of digital markers on the photograph by clicking in locations suggested by dynamic instructions. If needed, the markers are automatically connected by lines. Most of the measurements are based upon straightforward vector length computation. Level difference measurements need an additional step of horizontal projection of the digital markers onto the midline and then calculating the distance between the resulting coordinates. To allow precise point marking, BI functions are enhanced with photograph transformation capabilities—translation, scaling and rotation. The user can also adjust its brightness and contrast (Fig. 2).

**Fig. 3:**
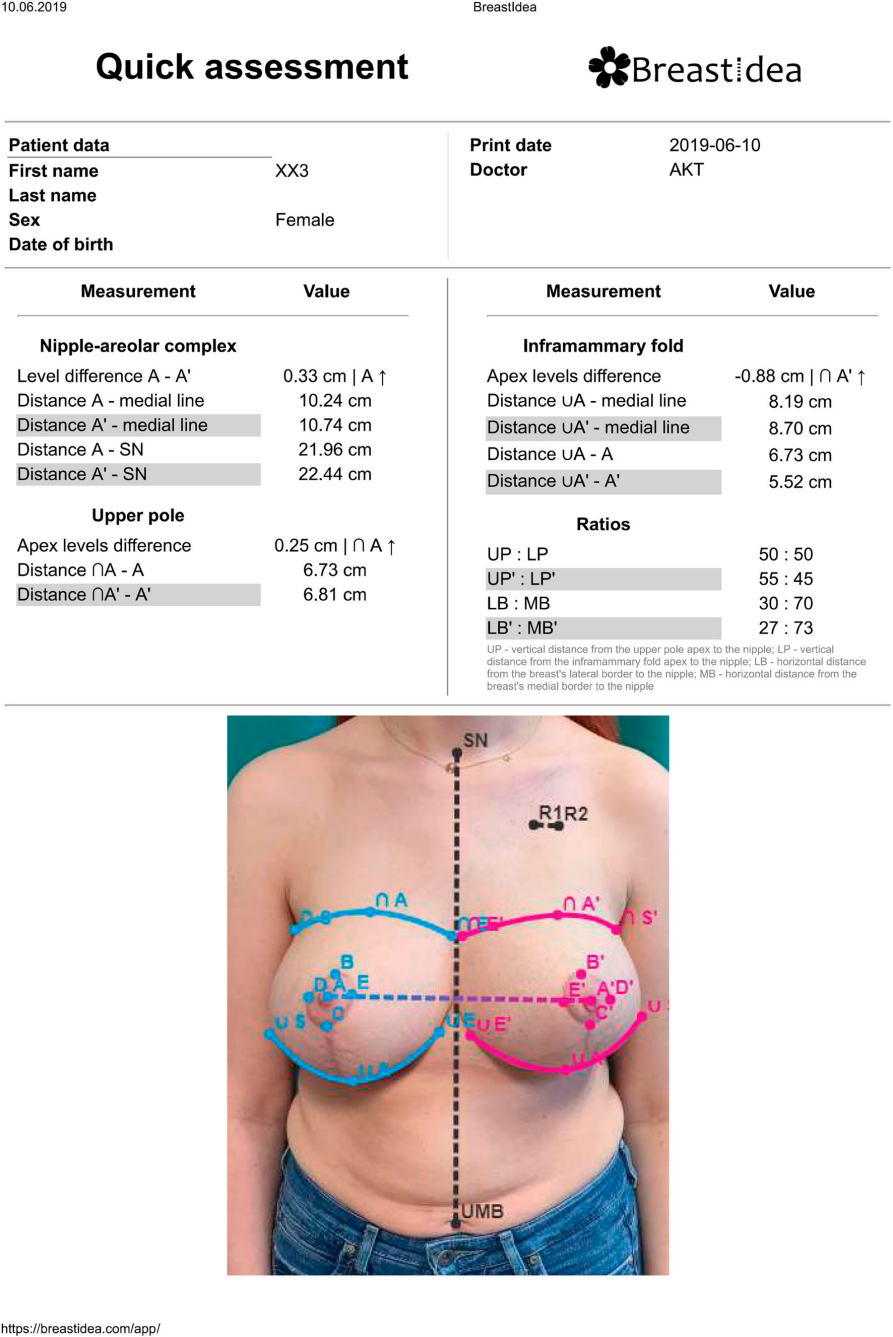
The report exported by the web application, with all the digital markers drawn on the subject’s photograph

In many patients, the upper breast border is not evident, especially on photographical data. To determine the breast footprint, the breast has to be lifted. Taking this into account, an option to use a second photograph was added. This option was designed especially for patients with breast ptosis—the patient is photographed while lifting their breasts to visualise inframammary folds and upper breast borders. This function makes it possible to correctly assess parameters referring to the inframammary fold and better depict the upper pole of the breasts. The two photographs are spatially bound together by marking the patient’s medial line on both of them. After all the measurements are made, BI generates a report with all the markers and lines present on the photograph, as well as essential patient information (Fig. 3). The report can then be printed or saved as a PDF file, providing an easy way to enclose the results of the breast assessment process in both physical and electronic medical documentation.

The application uses the web browser only as an environment to run JS logic. The photographs used in BI are not uploaded to the web. No data were sent out or fetched from a database—data were only handled locally in the browser. BI is available free of charge for everyone at www.breastidea.com/app.

### Direct Breast Measurements

Direct breast measurements took place at different times of day and in random lighting conditions. The surgeon placed a reference unit (2 cm long) on the chest, either below a clavicle or on the sternum, below the sternal angle. The location of the reference unit did not affect measurement accuracy, so optimal reference unit placement was dependent on the patient’s body shape, as long as the surface was flat and perpendicular to the camera. The unit was used to translate virtual length to real-world length. A digital photograph of the subject’s chest was taken, making sure the camera (We consider that most potential users will take pictures with their smartphones. However, because of security restrictions imposed by the Ethics Committee, we were not allowed to take photographs with a mobile phone. We made sure the digital camera parameters were as close to a standard smartphone’s camera as possible.) was perpendicular to the ground so as not to skew the perspective. Based on breast size and ptosis, the need for an additional photograph was evaluated. In those situations, the subject was asked to elevate their breasts for the second photograph.

Next, to acquire the reference data, all previously listed measurements were performed directly (gold standard method). All essential lines were drawn on the subject’s chest using a marker. Then, using a tape measure, the linear parameters were measured. To measure level differences, the right and left inframammary folds apices, upper pole apices and nipples were projected as points to the midline and the differences between these points were measured. The duration of measurements was noted. All measurements were done by one person (a plastic surgeon).

### Digital Indirect Breast Measurements

**Fig. 4:**
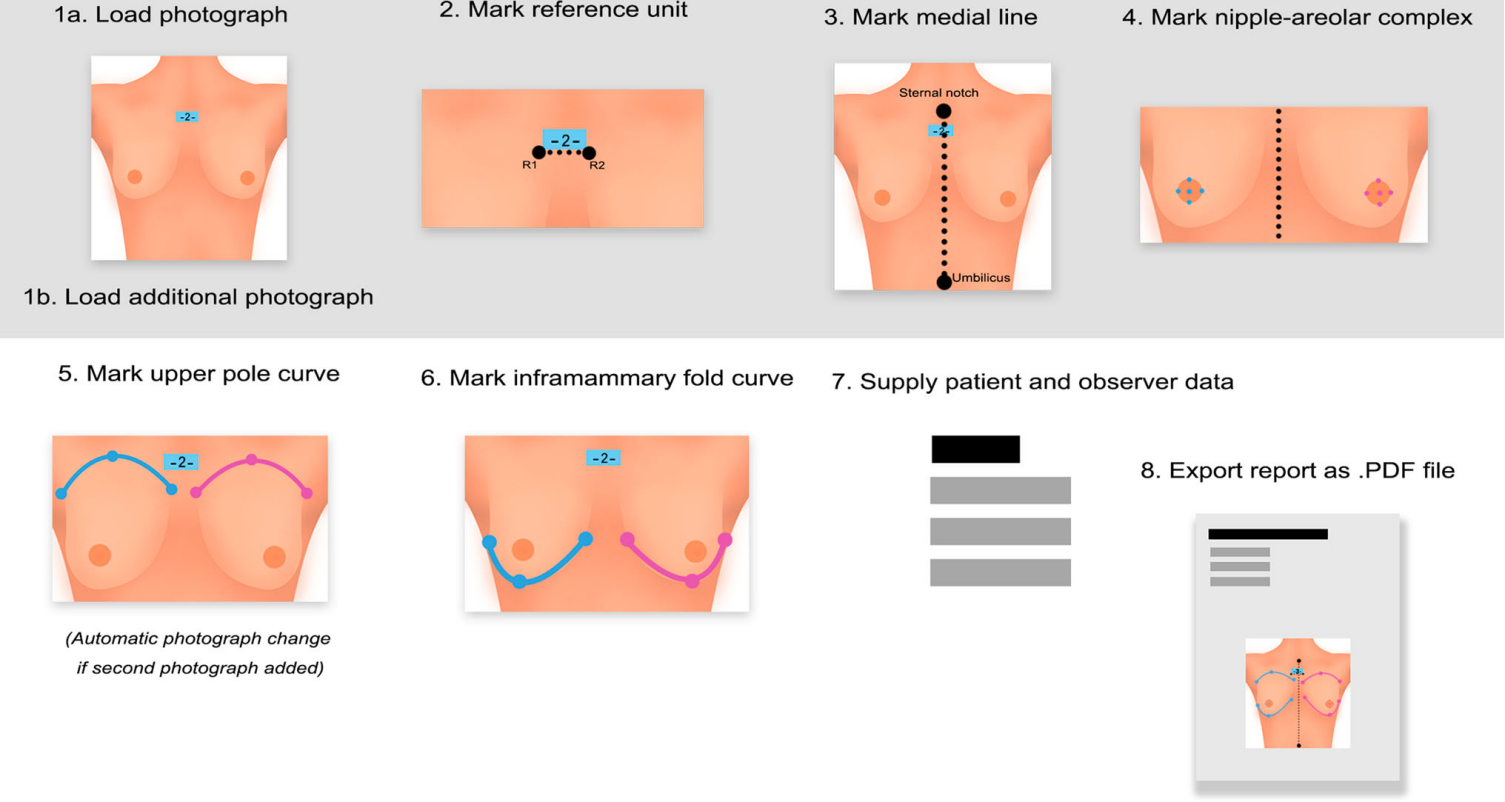
Application user experience flow concept

After transferring the photographs onto a computer, all measurements were made using BI (Fig. 4). The user is guided through the measurement process, which consists of placing digital markers by moving the cursor to indicated anthropometric landmarks and clicking the left mouse button. Measurements are automatically generated when necessary markers are placed. They are also updated in real time when the user adjusts the markers.

### Data Quality

To guarantee the quality of the data and their suitability we conducted the following procedures. Subjects were chosen randomly, regardless of breast shape and size. Direct measurements were made by one person (a plastic surgeon). Breast measurements using BI were performed independently by two researchers of different experience in breast measurements (a plastic surgeon other than the one collecting direct measurements and a medical student). Digital measurements on the subjects’ photographs were repeated at weekly intervals during three sessions, and a set of photographs was ordered randomly each time.

### Statistical Analysis

Accuracy (the degree of relation between the established ground-truth reference values measured physically and the corresponding digital measurements) was presented as mean absolute difference (MAD), i.e. expected absolute difference of two independent variables. Precision of measurement (variability of measurements performed by the same observer or relative reliability) for each variable was established using intra-class correlation coefficients (ICCs), i.e. a summary statistic describing resemblance of units between groups, and their 95% confidence intervals (95% CI). ICCs and coefficients of variation (CV), i.e. ratio of the standard deviation to the mean, were used to test absolute reliability (measurement independent of observer) [9]. Hopkins (2000) and Atkinson and Nevill (1998) strongly recommend providing more than one measure of reliability [10, 11]. Thus, ICCs were supplemented with standard error of measurement (SEM), i.e. the standard deviation of measurement errors, as this measure is more explicit in clinical context by giving results in the same units as the measurements [12]. The most common interpretation of ICC values follows Cicchetti’s guidelines: values in range 0.00–0.39 are considered poor correlation, 0.40–0.59—fair correlation, 0.60–0.75—good correlation and 0.75–1.00—excellent correlation [13]. A more recent clinically oriented interpretation suggests that values below 0.75 represent poor to moderate correlation, values between 0.75 and 0.87 are good correlation, and values above 0.87 represent correlation high enough to be regarded as clinical measures [14]. Accepted levels of measurement deviation for the thoracic region are below 1 cm [15]. Hence, such a range will be classified as good accuracy.

To show clinical reliability, the Bland–Altman plot was created, juxtaposing differences between values measured directly and in BI, and the mean of three measurements [16, 17]. Calculations were performed using Statistica v13 (Dell Inc., Round Rock, TX). *P* value ≤ 0.05 was considered as the threshold of statistical significance.

## Results

Accuracy and precision for both female and male subjects, as well as inter-rater variability, are shown in Tables 1, 2 and 3. Absolute differences between digital measurements and reference measurements lie below the 1-cm demarcation line. There are, however, accuracy drops for specific measurements (medial line–nipple distance).

**Table 1:** Summary of three-session validation for female subjects

Measurement	Observer A					Observer B				
	Accuracy		Precision			Accuracy		Precision		
	MAD (cm)	SD (cm)	ICC	95% CI	SEM (cm)	MAD (cm)	SD (cm)	ICC	95% CI	SEM (cm)
NLD	0.30	0.29	0.944	0.847–0.984	0.07	0.41	0.31	0.988	0.965–0.997	0.03
MLN	1.42	1.06	0.935	0.824–0.982	0.27	1.71	1.08	0.970	0.915–0.992	0.19
MLN*	1.91	1.21	0.930	0.812–0.980	0.32	1.61	1.03	0.958	0.883–0.988	0.21
SNN	0.11	1.38	0.960	0.889–0.989	0.28	0.15	1.07	0.977	0.935–0.994	0.16
SNN*	0.12	1.30	0.952	0.868–0.987	0.28	0.14	0.94	0.969	0.913–0.991	0.17
IMFLD	0.34	0.30	0.951	0.865–0.986	0.07	0.29	0.24	0.962	0.894–0.990	0.05
IMFML	0.39	0.99	0.935	0.824–0.982	0.25	0.26	1.04	0.926	0.802–0.979	0.28
IMF*ML	0.14	1.03	0.936	0.827–0.982	0.26	0.50	1.31	0.985	0.957–0.996	0.16
IMFN	0.32	1.25	0.843	0.617–0.954	0.50	0.53	1.51	0.948	0.857–0.986	0.34
IMF*N*	0.26	1.06	0.826	0.584–0.949	0.44	0.05	1.08	0.955	0.875–0.988	0.23
UPLD	0.57	1.44	0.905	0.752–0.973	0.45	0.70	1.33	0.932	0.817–0.981	0.35
UPN	0.58	1.18	0.992	0.977–0.998	0.11	0.68	1.00	0.988	0.965–0.997	0.11
UPN*	0.70	1.22	0.993	0.980–0.998	0.10	1.00	1.04	0.983	0.951–0.995	0.14

*MAD* mean absolute difference, *SD* standard deviation, *ICC* intra-class correlation coefficient, *CI* confidence interval, *SEM* standard error of measurement, * left, *NLD* nipple levels difference, *MLN* medial line to nipple distance, *SNN* sternal notch to nipple distance, *IMFLD* inframammary fold apex levels difference, *IMFML* inframammary fold apex to medial line distance, *IMFN* inframammary fold to nipple distance, *UPLD* upper pole apex levels difference, *UPN* upper pole apex to nipple distance

**Table 2:** Summary of three-session validation for male subjects

Measurement	Observer A					Observer B				
	Accuracy		Precision			Accuracy		Precision		
	MAD (cm)	SD (cm)	ICC	95% CI	SEM (cm)	MAD (cm)	SD (cm)	ICC	95% CI	SEM (cm)
NLD	0.36	0.20	0.887	0.711–0.968	0.07	0.31	0.18	0.953	0.870–0.987	0.04
MLN	0.47	0.46	0.986	0.960–0.996	0.05	0.62	0.47	0.996	0.988–0.999	0.03
MLN*	0.21	0.40	0.984	0.954–0.996	0.05	0.33	0.39	0.994	0.983–0.998	0.03
SNN	0.89	0.84	0.973	0.924–0.993	0.14	1.12	0.75	0.994	0.983–0.998	0.06
SNN*	0.74	0.77	0.973	0.924–0.993	0.13	0.90	0.68	0.991	0.974–0.998	0.06
IMFLD	0.27	0.22	0.909	0.761–0.974	0.07	0.26	0.22	0.950	0.862–0.986	0.05
IMFML	0.32	0.50	0.987	0.963–0.996	0.06	0.27	0.43	0.993	0.980–0.998	0.04
IMF*ML	0.09	0.59	0.972	0.921–0.992	0.10	0.07	0.54	0.976	0.932–0.993	0.08
IMFN	0.15	0.34	0.988	0.965–0.997	0.04	0.23	0.34	0.990	0.971–0.997	0.03
IMF*N*	0.37	0.38	0.979	0.940–0.994	0.06	0.41	0.41	0.997	0.991–0.999	0.02
UPLD	0.16	0.35	0.963	0.897–0.990	0.07	0.27	0.29	0.959	0.886–0.989	0.06
UPN	0.11	0.34	0.991	0.974–0.998	0.03	0.20	0.28	0.998	0.994–0.999	0.01
UPN*	0.14	0.32	0.996	0.988–0.999	0.02	0.29	0.31	0.999	0.997–1.000	0.01

*MAD* mean absolute difference, *SD* standard deviation, *ICC* intra-class correlation coefficient, *CI* confidence interval, *SEM* standard error of measurement, * left, *NLD* nipple levels difference, *MLN* medial line to nipple distance, *SNN* sternal notch to nipple distance, *IMFLD* inframammary fold apex levels difference, *IMFML* inframammary fold apex to medial line distance, *IMFN* inframammary fold to nipple distance, *UPLD* upper pole apex levels difference, *UPN* upper pole apex to nipple distance

**Table 3:** Summary of intra-observer reliability between Observer A and Observer B, based on average values from three sessions

Measurement	Female subjects				Male subjects			
	CV (%)	ICC	95% CI	SEM (cm)	CV (%)	ICC	95% CI	SEM (cm)
NLD	5.7	0.968	0.910–0.991	0.01	6.3	0.943	0.844–0.984	0.01
MLN	1.2	0.968	0.910–0.991	0.02	0.7	0.996	0.988–0.999	0.00
MLN*	0.1	0.924	0.797–0.979	0.00	0.5	0.998	0.994–0.999	0.00
SNN	0.1	0.989	0.968–0.997	0.00	0.6	0.996	0.988–0.999	0.01
SNN*	0.0	0.986	0.960–0.996	0.00	0.4	0.997	0.991–0.999	0.00
IMFLD	5.8	0.909	0.761–0.974	0.01	0.4	0.957	0.881–0.988	0.00
IMFML	2.2	0.953	0.870–0.987	0.04	0.2	0.992	0.977–0.998	0.00
IMF*ML	3.0	0.858	0.648–0.959	0.11	0.1	0.995	0.985–0.999	0.00
IMFN	4.5	0.810	0.553–0.943	0.15	0.9	0.998	0.994–0.999	0.00
IMF*N*	1.2	0.898	0.736–0.971	0.04	0.9	0.995	0.985–0.999	0.01
UPLD	4.9	0.944	0.847–0.984	0.09	0.5	0.967	0.907–0.991	0.00
UPN	2.3	0.987	0.963–0.996	0.02	0.6	0.998	0.994–0.999	0.00
UPN*	3.8	0.952	0.868–0.987	0.08	1.0	0.999	0.997–1.000	0.00

*CV* coefficient of variation, *ICC* intra-class correlation coefficient, *CI* confidence interval, *SEM* standard error of measurement, * left, *NLD* nipple levels difference, *MLN* medial line to nipple distance, *SNN* sternal notch to nipple distance, *IMFLD* inframammary fold apex levels difference, *IMFML* inframammary fold apex to medial line distance, *IMFN* inframammary fold to nipple distance, *UPLD* upper pole apex levels difference, *UPN* upper pole apex to nipple distance

Precision (consistency of measurement) as well as inter-rater reliability, is at clinical measures level (or excellent according to Cichetti) for all but two measurements (female subjects' right inframammary fold apex to right nipple distance, which was 0.060 below the limit and left inframammary fold to medial line distance, 0.012 below the limit). Precision ICC values have a larger variance for female subjects (0.001415) than for male subjects (0.000705). Absolute reliability ICC values also have a larger variance for female subjects (0.00290) when compared to male subjects (0.0003465).

Standard error of measurement is consistent with ICC values. It is mostly below 0.1 cm for female subjects and close to zero for male subjects. Coefficient-of-variation values are the highest (around 5–6%) for level differences of nipples, inframammary fold apices and upper pole apices.

**Fig. 5:**
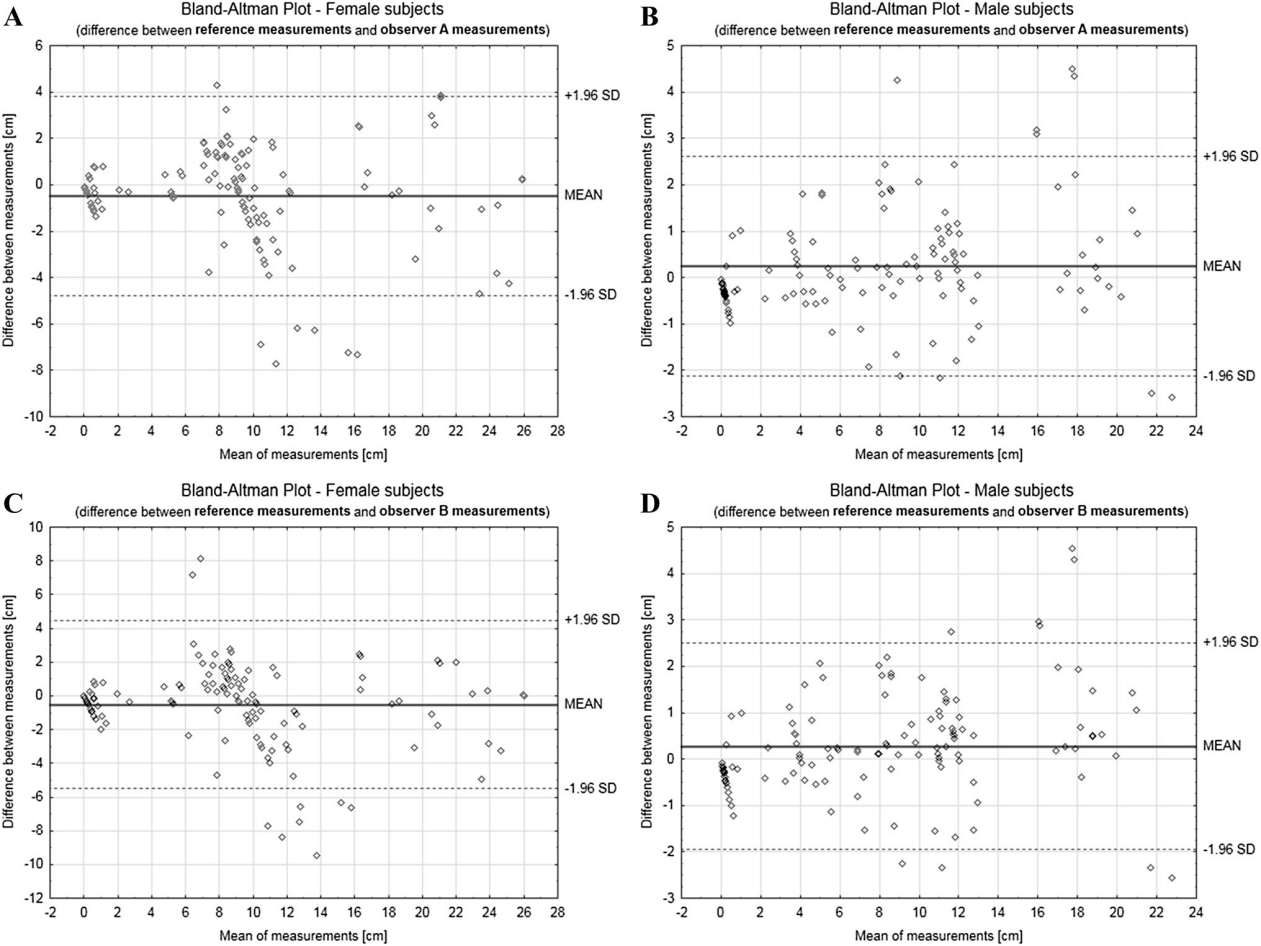
Bland–Altman plots, presenting the difference between reference measurements and: observer A digital measurements for female (**a**) and male (**b**) subjects; observer B digital measurements for female (**c**) and male (**d**) subjects

The Bland–Altman plots (Fig. 5a–d) show how the overall data map out when juxtaposed with the reference data (direct measurements).

The effectiveness (probability of satisfactory result) of BI equals 87.5% for females and 95.8% for males.

Breast assessment using BI during the first measurement session took around 1.5 min (with an eight-second difference when two photographs were used) for females and about a minute for males. The average times in the last session are roughly 30% shorter for female subjects and 20% shorter for male subjects (Table 4).

**Table 4:** Summary of measurements average duration

	Female subjects		Male subjects
	Average (min)		Average (min)
	1 photograph	2 photographs	
Session 1	1.43 ± 0.28	1.56 ± 0.22	0.97 ± 0.09
Session 2	1.21 ± 0.25	1.41 ± 0.25	0.83 ± 0.10
Session 3	0.96 ± 0.18	1.15 ± 0.09	0.82 ± 0.09

## Discussion

The study presents validation of BI—software dedicated to indirect breast anthropometry. It was designed for clinical purposes; however, it can also be helpful in scientific analyses. The process of validation included male and female participants, regardless of breast anatomy, which mimics the variety of plastic surgeons’ patients. The measurements performed by two examiners of different clinical experience using BI were compared to ‘gold standard’ direct measurements performed by a plastic surgeon. High consistency of the results suggests high repeatability of measurements independent of examiner’s experience.

As proved by Odo et al. [18], direct anthropometry is a valid method to depict differences between breasts when comparing asymmetry before and after surgery [18]. However, direct measurements have several drawbacks. While some body regions can be assessed in a mostly linear manner, the chest has prominent curvatures, making measurements less accurate [2, 19, 20]. Also, the region is not only affected by whole-body movements, but also ribcage expansion while breathing. Indirect anthropometry on the other hand presents a wide array of advantages. According to numerous studies, not only does it allow higher reliability, but it is also much less stressful for the patient [19, 21–23]. Data are also usually relatively easy to record, already being in digital format. Nonetheless, indirect anthropometry of the breasts based on digital photographs using standard software also fails in some aspects. The programs offer satisfactory precision and accuracy only for a specific group of patients [5]. For example, for women with ptotic breast these programs fail to produce highly reliable results. Adapting programs like Autodesk AutoCAD^®^ or Adobe Photoshop^®^ for such use imposes the need to train medical staff and create a specific list of commands, resulting in a nonlinear workflow [5]. The programs are vast packages, offering a number of tools needed in the industry they were designed for, while in clinical practice their capabilities can prove daunting and distracting for the user [24, 25].

The middle ground between direct anthropometry and measurements using two-dimensional photographs is photogrammetry [26]. It is a valid approach; however, producing stable, good results requires a lot of experience, especially for patients with severe breast ptosis. Another drawback is the need for lengthy computation using a high-end computer or external cloud service (unadvised for sensitive data) [27].

In this paper, we presented the self-designed tool (and its validation), which does not require a lot of time from a user to become confident in usage, being as well capable of running on any modern device without specific hardware requirements. Because of the natural differences between male and female chests, it was crucial to validate the tool in female and male groups. We found that most accuracy values for female subjects vary by ± 1 cm, consistently between observers. The exceptions are nipple level differences and inframammary fold apex level differences. Their standard deviation was around 0.30 cm. Standard deviation of mean absolute difference above 1 cm was not detected in measurements in males. BI measurements, which were juxtaposed with measurements mostly affected by curves in direct anthropometry are least accurate, with a mean absolute difference between 1.42 and 1.91 cm (medial line–nipple distance). This may result from the fact that for distances like the one traced from the nipple to the body’s medial line, one should demarcate a line segment tangent to the natural breast curve, which is not a routine approach in clinical breast measurements [2]. It appeared that BI gives valid results when compared to a textbook procedure (measuring straight lines instead of following natural curves) [2]. The discussed problem is also depicted in the Bland–Altman graphs, where the majority of points fall between the two limits, proving suitability for clinical use, with a cluster of outlying data present for female subjects for both observers, corresponding to the measurements listed above.

To properly evaluate BI’s accuracy, we need to consider measurements which were truly linear both in the direct and indirect anthropometry using the web application. These measurements (sternal notch to nipple distance, medial line to inframammary fold apex distance, upper pole apex to nipple distance) mostly present satisfactory accuracy with excellent correspondence between observers. MAD of the distance from the inframammary fold apex to the medial line was initially above 2 cm, but decreased to about 0.30 cm within the next two sessions. What is more, the average accuracy of both observers grew with each session, accompanied by a significant decrease in procedure duration and time variation between different subjects. In the case of the distance from the inframammary fold apex to the medial line, we suppose the progress can be attributed to better visual extrapolation of anthropometric landmarks—applying experience from assessing subjects, whose specific points were more distinct, to female subjects with breasts that are more difficult to assess. This would also explain why there was no accuracy increase for male subjects—their chests were much easier to measure correctly.

For both male and female subjects, nearly all the ICC values fall above 0.87 and can be considered clinical measures—most reach at least 0.93. Reliability of direct anthropometry lies between 0.03 and 0.25 cm [28]. The SEM values both for inter and intra-rater reliability prove BI’s reliability is higher. Manual measurements usually have a coefficient of variation of 5% or more [28]. Although this percentage is still acceptable for the thoracic region, measurements with BI, on average, scored less than 2.7% for female subjects and 1.0% for male subjects.

When compared to graphics software adapted for breast measurements, BI’s accuracy is similar. However, mean absolute differences for female breasts obtained using BI generally deviate more when referred to standard deviation values presented by Quieregatto et al., what may be attributed to the fact, that their direct anthropometry measurements were made using callipers (truly linear measurement), while we used a tape measure and introduced curvature bias [5]. Analysing BI in comparison to 3D scanners, it should be highlighted that low- to medium-tier 3D scanners provide a reliability comparable to manual methods [29–31]. When comparing BI to the 3D scanner used by Conkle et al., the accuracy results for linear measurements are similar, while reliability outcomes are somewhat better [24]. On the other hand, BI’s accuracy is hindered by pixel resolution and is ten times lower than accuracy of an Artec Eva high-precision laser 3D scanner (Artec, Luxembourg) used by Seminati et al., while achieving comparable reliability [32].

What is more, even though we designed the application with ease of use as our top priority, we did not manage to avoid a learning curve for the user. While precision did not show a major change during the three sessions, accuracy and measurement time improved. The progress was noticeable for the assessment of female breasts.

Last but not least, striving for aesthetic breast assessment, we have used ratios proposed by Mallucci et al. and Lewin et al. [7, 8]. When performing direct measurements, the clinical practitioner had to calculate those proportions themselves. In BI the ratios are automatically calculated, saving the observer some effort.

One of the main limitations of the study was the number of participants. We decided ten males and ten females were sufficient to validate the application, especially when the stated data quality procedures were taken into account. The problem was tackled by random choice of subjects, resulting in a wide array of different breast shapes and sizes. However, this may also have resulted in data bias, so further study with a larger sample of patients should be performed. It is possible that separate validations should have been conducted for different breast deformations. Further limitation is related to the specificity of using indirect anthropometry—the technique that cannot address skin extensibility. On the other hand, skin extensibility makes direct measurement dependent on the technique of taking the measurements by certain surgeons, which is overcome by standardised indirect technique.

## Conclusion

To conclude, we found that BI may be a useful tool in breast surgeon’s clinical practice. It provides an objective method of breast measurement, offers high accuracy and precision without the need to process 3D data and omits the need for drawing dots and lines on the patient’s body. The user can accurately assess asymmetry, not being limited by chest deformations, severe ptosis or the necessity to take photographs in specific conditions. Finally, by using a digital medium, breast measurements no longer have to be written on physical paper or typed in a spreadsheet, while the report generated by BI may help analyse the treatment plan and consult it with another specialist regardless of the patient’s presence.
